# A multilevel analysis of neighborhood and individual effects on individual smoking and drinking in Taiwan

**DOI:** 10.1186/1471-2458-7-151

**Published:** 2007-07-10

**Authors:** Ying-Chih Chuang, Yu-Sheng Li, Yi-Hua Wu, Hsing Jasmine Chao

**Affiliations:** 1 Graduate Institute of Public Health, Taipei Medical University, 250 Wu-Hsing St., Taipei, Taiwan

## Abstract

**Background:**

We assessed direct effects of neighborhood-level characteristics and interactive effects of neighborhood-level characteristics and individual socioeconomic position on adult smoking and drinking, after consideration of individual-level characteristics in Taiwan.

**Methods:**

Data on individual sociodemographic characteristics, smoking, and drinking were obtained from Taiwan Social Change Survey conducted in 1990, 1995, and 2000. The overall response rate was 67%. A total of 5883 women and men aged over 20 living in 434 neighborhoods were interviewed. Participants' addresses were geocoded and linked with Taiwan census data for measuring neighborhood-level characteristics including neighborhood education, neighborhood concentration of elderly people, and neighborhood social disorganization. The data were analyzed with multilevel binomial regression models.

**Results:**

Several interaction effects between neighborhood characteristics and individual socioeconomic status (SES) were found in multilevel analyses. Our results indicated that different neighborhood characteristics led to different interaction patterns. For example, neighborhood education had a positive effect on smoking for low SES women, in contrast to a negative effect on smoking for high SES women. This result supports the hypothesis of "relative deprivation," suggesting that poor people living in affluent neighborhoods suffer from relative deprivation and relative standing. On the other hand, neighborhood social disorganization has positive effects on drinking for low SES individuals, but not for high SES individuals. These interactive effects support the hypothesis of the double jeopardy theory, suggesting that living in neighborhoods with high social disorganization will intensify the effects of individual low SES.

**Conclusion:**

The findings of this study show new evidence for the effects of neighborhood characteristics on individual smoking and drinking in Taiwan, suggesting that more studies are needed to understand neighborhood effects in Asian societies.

## Background

Smoking and drinking are highly prevalent in Taiwan among male adults, despite the accumulated evidence on the serious health and safety consequences [1-4]. According to the 2002 Taiwan National Health Knowledge, Attitude and Practice Interview Survey, the daily smoking rates were 43.5% and 4.2% for men and women, respectively. About 53.1% of men and 23.1% of women reported drinking frequently, while 24.6% of men and 7.9% of women reported having problem drinking behaviors [5]. Although women consistently show lower rates of smoking and drinking than men, the prevalence rates of these behaviors are increasing due to greater economic independence and changes in social concepts [6]. Although there are only 23 cities and counties in Taiwan, the 23 cities and counties showed distinctively wide disparities in smoking and drinking behaviors [7]. One of the possible explanations comes from local norms and sanctions that may inhibit or promote one's smoking and drinking behaviors suggesting examining these behaviors from a neighborhood-level perspective.

Prior studies have suggested that neighborhood-level characteristics have independent effects on individual-level smoking and drinking behaviors after consideration of individual-level socioeconomic status/position (SES) [8-25]. There are several postulated reasons why neighborhood characteristics may influence individual smoking and drinking. These include social norms, psychosocial stress, exposure to tobacco and alcohol advertising, and availability of tobacco and alcohol [10,26].

One major cited criticism on prior studies is the lack of examination of cross-level interactions between neighborhood characteristics and individual socioeconomic status [11,27,28]. Most researchers "average" the effects of neighborhood-level variables across individuals, despite some evidence that neighborhood effects may be heterogeneous across different individual socioeconomic positions [29]. Two possible hypotheses may be generated from the interaction of neighborhood-level characteristics and individual-level SES. The first one is the "double jeopardy" hypothesis, which suggests that the harmful effects of individual poverty could be intensified for those who live in poor neighborhoods [30]. On the other hand, the "relative deprivation" hypothesis suggests that poor people living in affluent neighborhoods may be stressed from perceived income inequality and thus suffer from relative deprivation and relative standing [30-32]. Only a handful of studies, to our knowledge, have examined how neighborhood effects interacted with individual SES. More studies supported the hypothesis of double jeopardy, while the other studies supported the relative deprivation hypothesis or found no interaction effects of neighborhood characteristics and individual SES [33-39].

Despite an increasing number of studies focusing on neighborhood influences on individual health outcomes, few studies were conducted in Asian societies. Neighborhood effects may be drastically different between Asian and Western societies due to the fundamental differences in social relationships, community formation, and economic development [40]. Our study contributes to the understanding of neighborhood-level influences on individual smoking and drinking behaviors in Taiwan. We also examine whether neighborhood influences on individual smoking and drinking behaviors depend on individual socioeconomic position and which hypothesis (double jeopardy vs. relative deprivation) is supported.

## Methods

### Data

The individual-level data are from the 1990, 1995, and 2000 Taiwan Social Change Survey, which is a repeated cross-sectional study conducted every 5 years [41-43]. A multi-stage cluster sampling method was used to select adults aged over 20 for the survey. It first divided 359 township/districts of Taiwan into ten strata according to geographic location and degree of urbanization. Townships or districts in each stratum were selected by probability proportional to their size (PPS). In each selected township/district, lis and villages were selected by PPS and individuals were randomly selected in lis and villages. Lis and villages are small geographical units created by the Taiwan Census Bureau for studying neighborhoods. The size of a Li is smaller than a census tract but larger than a census block group in US. Each li has on average 2000 people and 874 households. Data were collected by interpersonal interviews using a structured questionnaire. Interviewers were required to attend a standardized 2-day training workshop before conducting interviews. The overall response rate was 67% after excluding ineligible cases. The major reasons for not completing the interview included an inability to find the person (18.3%) and refusal to participate (11.2%). Ten percent of the cases were rechecked for quality control. This study defines neighborhoods by lis and villages. They were created by visible boundaries such as streets and rivers and to be as homogeneous as possible with population characteristics. Participants' residential addresses were geocoded with 1990 and 2000 Taiwan census data; linear interpolation was used for the 1995 data. Six percent of the respondents were not accurately geocoded to their neighborhoods based on home address, resulting in a final sample size of 434 neighborhoods and 5,883 people. Informed consent was obtained from each participant. The ethical committee of Taiwan National Science Council approved this study.

### Dependent variables

Individual-level smoking was measured from the question, "On average, about how many cigarettes do you now smoke in a day?" with responses ranging from "no cigarettes" to "more than two packs a day" along a 7-point scale. Because prior research suggested that disadvantaged neighborhood characteristics were associated with the likelihood of being a current smoker, smoking was recoded as 0 if no use of cigarette and 1 if use of cigarette. Individual-level drinking was measured from the question, "How often do you drink alcohol?" along a 4-point scale including "none at all," "occasionally," "drink often, rarely get drunk," and "drink often, often get drunk". Drinking was recoded as 0 if no use of alcohol and 1 if use of alcohol. Most prior studies focusing on neighborhood influences on individual drinking used problem drinking behavior as the outcome of interest. However, our data contain very few problem-drinking cases, less than one percent of the participants identified themselves as drinking frequently and often getting drunk. Therefore we were unable to examine the effects of neighborhood characteristics on problem drinking. In addition, some studies suggested that the effects of neighborhood characteristics on drinking may vary according to different levels of alcohol intake. For example, studies found that neighborhood-level social capital was more likely to be associated with moderate drinking, compared to no drinking and heavy drinking, indicating that neighborhood-level social activities were more likely to stimulate a moderate intake of alcohol [44]. This suggests each level of alcohol drinking may have its own theoretical meaning. We categorized drinking behaviors into "use of alcohol" and "not use of alcohol", which can compare the differential neighborhood effects between individuals who decide not to drink or does not exposure to any alcohol versus the others.

### Individual-level variables

Individual-level SES was calculated from two indicators: educational attainment and monthly household income. Education was measured by asking respondents, "What is the highest level of formal education you have completed?" with responses ranging from "Lower than elementary school" to "Graduate School" on a 7-point scale. Income was measured by asking participants, "How much is your household's total income per month, including income from all sources for all household members living with you?" with responses ranging from "under NT$10, 000" to "NT$220,000 and over" on a 7-point scale (1 US Dollar = 33 New Taiwan Dollars). A composite SES score was created by averaging levels of education and family income for each respondent. The score of SES was categorized into "high" versus "low" using a median split. Gender, age (20–39, 40–59, ≥ 60), race/ethnicity (Taiwanese, Hakka, Mainlanders, indigenous populations, and others), marital status (single, married, divorced and separated, and others), and year of surveys (1990, 1995, and 2000) were included in the analyses as control variables. We categorized age into three categories (20–39, 40–59, ≥ 60) in order to both consider the power of analysis and the differential impacts of neighborhood characteristics on people in different life stages (adults before mid-age, mid-age, and elderly). Because more than 70% of people were Taiwanese, we created a dummy-coded variable and used non-Taiwanese as the reference group. Marital status was recoded as 1 = married and 0 = others to measure the social support in marriage.

### Neighborhood-level variables

Nine neighborhood-level indicators were derived from 1990 and 2000 census data; linear interpolation was used for the 1995 data. These variables were selected based on previous theoretical and empirical neighborhood research [45-50]. Because neighborhood measurements in Taiwan are still under development, we conducted an exploratory factor analysis and used factor scores to represent neighborhood domains (Table 1) [51]. Three factors were identified, including neighborhood education, neighborhood concentration of elderly people, and neighborhood social disorganization. The two items of residential mobility neither formed a single factor nor loaded well on other factors. Therefore, we disregarded the 2 items in further analyses. Neighborhood education was measured by two indicators: (1) percentage of less than junior high school and (2) percentage of college graduates (Cronbachα = .90) with a higher score representing a higher neighborhood education. Neighborhood concentration of elderly people was measured by two indicators: (1) percentage of age under 18 and (2) percentage of age over 65 (Cronbachα = .75) with a higher score representing a higher concentration of elderly people. Neighborhood social disorganization was measured by three indicators: (1) percentage of paid employment (2) percentage of divorced and separated, and (3) percentage of single-parent families (Cronbachα = .52) with a higher score representing a higher neighborhood social disorganization. The correlation coefficients for the three neighborhood domains ranged from 0.07 to 0.25 suggesting they were weakly to moderately correlated. In addition to the seven neighborhood characteristics, locality was introduced as a control variable measured by the proportion of people who live in rural, suburban, or urban areas.

**Table 1:** Factor analysis of neighborhood characteristics, Taiwan census data, 1990, 1995, and 2000

	Neighborhood education	Neighborhood concentration of elderly people	Neighborhood social disorganization
% less than junior high	***0.95***	-0.08	-0.01
% college graduate	***-0.96***	-0.04	-0.03
% less than age 18	0.25	***0.86***	0.03
% more than age 65	0.22	***-0.89***	0.07
% single parent family	-0.11	0.39	***0.65***
% divorced and separated	-0.31	-0.23	***0.58***
% paid employment	-0.18	0.04	***-0.82***

### Analysis

We used multilevel models to analyze our data. We used the SAS macro GLIMMIX to fit multilevel models with a binomial distribution assumption and a logit link. The method of estimation was a restricted maximum likelihood procedure. Models were first fitted with neighborhood-level characteristics. The second stage was to fit models with individual-level characteristics, which were selected based on prior literature. In the third stage, models included both neighborhood-level and the significant individual-level characteristics identified in the second stage to assess whether neighborhood-level effects were explained by individual characteristics. Lastly, two-way interaction terms of individual-level SES and separate neighborhood-level characteristic were added to the models to test whether the effects of neighborhood-level characteristics on smoking and drinking were modified by individual-level SES. All analyses were conducted separately by gender.

## Results

Table 2 presents descriptive statistics for key variables by gender. In women, about half of the respondents in the sample were aged between 30 and 49. A large majority was married at the time. The largest racial/ethnic group was Taiwanese. About half of the sample had completed junior high school, which is the highest compulsory education in Taiwan, and about 70% of the sample had incomes over NT30, 000. Half of the sample lived in suburban areas. Because we stratified neighborhood-level characteristics into tertiles based on the distribution in each year, each stratum accounted for approximately one-third of the sample. Similar patterns were found for men, except that a higher percentage of men had completed college. Neighborhood characteristics were similar for women and men. Standard deviation was greatest for the percentage of less than junior high school and smallest for the percentage of divorced and separated.

**Table 2:** Individual-level characteristics and neighborhood-level characteristics by gender, Taiwan Social Change Survey, 1990, 1995, and 2000

	Women	Men
	(N = 2942)	(N = 2941)
Individual characteristics (%)		
Age		
20–29	19.5	16.6
30–39	33.4	29.1
40–49	23.4	24.1
50–59	11.6	13.2
>= 60	12.1	17.0
Marital status		
Single	14.6	22.3
Married	73.1	70.9
Widowed/divorced and others	12.3	6.8
Race/ethnicity		
Taiwanese	72.7	69.4
Hakka	13.0	13.5
Mainlander	11.1	15.3
Indigenous and others	3.2	1.8
Education		
< Elementary	12.2	4.9
Elementary	27.9	24.0
Junior high school	13.6	17.5
High school	27.3	26.9
>= College	19.0	26.7
Family income		
<NT30,000	29.9	28.4
NT30,000–NT49,999	25.8	24.4
NT50,000–NT69,999	16.9	16.4
NT70,000–NT99,999	13.1	14.8
≥ NT100,000	14.3	16.0
Year		
1990	34.0	31.8
1995	34.0	35.8
2000	32.0	32.4
Urbanicity/rurality		
Urban	32.0	31.4
Suburban	49.0	49.4
Rural	19.0	19.2
Neighborhood characteristics (mean (SD))		
% less than junior high	37.7(14.2)	38.3(14.3)
% college graduates	7.5(7.0)	7.2(6.7)
% less than 18	28.8(5.4)	28.5(5.3)
% over 65	7.9(4.1)	8.1(4.3)
% single parent family	6.3(2.4)	6.2(2.4)
% divorced and separated	2.7(1.2)	2.7(1.2)
% paid employment	56.3(6.9)	56.3(7.0)

Multilevel modeling results are shown in Table 3 for smoking. Models 1 to 3 are random intercept models in which the mean of the outcome is varied by neighborhood. Model 4 is a random slope model in which the coefficient for individual SES was allowed to vary by neighborhood. For women, Model 1 indicates that the respondents in neighborhoods with higher social disorganization were more likely to smoke (OR = 1.34). Model 2 shows that women characterized as higher SES, living in rural areas, Taiwanese, and married reported a lower probability of smoking than their counterparts. Model 3 shows that the effects of neighborhood social disorganization disappeared after adjusting for locality and individual-level characteristics. However, after including interactions between individual SES and neighborhood-level characteristics in Model 4, the effects of neighborhood social disorganization reappeared (OR = 1.36). Model 4 also shows that individual SES was significantly interacted with neighborhood education (OR = 0.61).

**Table 3:** Associations between individual-level characteristics, neighborhood-level characteristics, and individual smoking (odds ratios), Taiwan Social Change Survey, 1990, 1995, and 2000

	Women (N = 2942)				Men (N = 2941)			
	
	Model 1	Model 2	Model 3	Model 4	Model 1	Model 2	Model 3	Model 4
Neighborhood education	1.03		0.95	1.25	0.78**		0.83**	0.85
	(0.85–1.25)^a^		(0.74–1.23)	(0.90–1.73)	(0.72–0.84)		(0.73–0.93)	(0.69–1.01)
Concentration of elderly people	0.89 (0.80–1.09)		0.87 (0.70–1.09)	0.98 (0.74–1.28)	0.94 (0.87–1.01)		0.95 (0.87–1.03)	0.92 (0.79–1.04)
Neighborhood social disorganization	1.34** (1.11–1.63)		1.23 (0.99–1.54)	1.36* (1.04–1.76)	1.02 (0.95–1.11)		1.03 (0.95–1.12)	1.13* (1.00–1.25)
Individual SES		0.67**	0.69**	0.38**		0.59**	0.63**	0.63**
		(0.37–0.96)	(0.53–0.90)	(0.25–0.57)		(0.42–0.76)	(0.46–0.81)	(0.45–0.80)
Suburban/urban		0.87	0.93	0.98		1.21*	1.06	1.04
		(0.43–1.30)	(0.54–1.58)	(0.52–1.87)		(1.04–1.38)	(0.86–1.26)	(0.83–1.24)
Rural/urban		0.51*	0.64	0.61		1.46*	1.02	1.02
		(-0.11–1.13)	(0.47–1.50)	(0.22–1.71)		(1.04–1.49)	(0.71–1.33)	(0.70–1.34)
Age 40–59/age 20–39		1.06				0.67**	0.67**	0.68**
		(0.76–1.35)				(0.49–0.85)	(0.50–0.84)	(0.50–0.85)
Age 60+/age 20–39		0.91				0.48**	0.50**	0.50**
		(0.50–1.31)				(0.24–0.72)	(0.26–0.73)	(0.27–0.74)
Taiwanese		0.30**	0.31**	0.28**		0.98		
		(0.01–0.60)	(0.23–0.42)	(0.21–0.37)		(0.78–1.19)		
Married		0.28**	0.29**	0.23**		0.94		
		(0.03–0.54)	(0.23–0.37)	(0.18–0.29)		(0.77–1.12)		
Year 1990/year 2000		1.50				1.53**	1.53**	1.55**
		(0.99–2.00)				(1.34–1.72)	(1.34–1.72)	(1.35–1.74)
Year 1995/year 2000		1.36				1.17	1.16	1.18
		(0.82–1.90)				(0.99–1.35)	(0.98–1.34)	(1.00–1.36)
Neighborhood education × individual SES				0.61* (0.41–0.89)				0.95 (0.77–1.13)
Concentration of the elderly people × individual SES				0.78 (0.52–1.17)				1.06 (0.91–1.22)
Neighborhood social disorganization × individual SES				0.88 (0.60–1.29)				0.85* (0.69–1.01)

*P < 0.05 **P < 0.01 ^a^95% confidence interval

For male smoking, Model 1 shows that individuals in higher educated neighborhoods were less likely to smoke (OR = 0.78). Men characterized as lower SES, living in suburban and rural areas, younger, and interviewed in 1990 had a higher probability of smoking than their counterparts (Model 2). Model 3 shows that neighborhood education remained negatively associated with individual smoking after including control variables (OR = 0.83). Although effects of neighborhood education disappeared after adding interaction effects, neighborhood social disorganization was positively associated with male smoking in Model 4 (OR = 1.13) and this relationship was modified by individual-level SES (OR = 0.85).

Table 4 presents a similar set of multilevel models for drinking. Model 1 for females shows that women in higher educated neighborhoods were more likely to drink (OR = 1.27). Women characterized as higher SES, younger, non-Taiwanese, and interviewed in 1990 were more likely to drink. The effects of neighborhood education remained significant after controlling for locality and individual characteristics (Model 3, OR = 1.16) and significant interactive effects were found between individual SES and concentration of elderly people (Model 4, OR = 1.27) as well as neighborhood social disorganization (Model 4, OR = 0.81).

**Table 4:** Associations between individual-level characteristics, neighborhood-level characteristics, and individual drinking (odds ratios), Taiwan Social Change Survey, 1990, 1995, and 2000

	Women (N = 2942)				Men (N = 2941)			
	
	Model 1	Model 2	Model 3	Model 4	Model 1	Model 2	Model 3	Model 4
Neighborhood education	1.27**		1.16**	1.02	1.15**		1.08	1.00
	(1.15–1.41)^a^		(1.05–1.26)	(0.85–1.19)	(1.04–1.27)		(0.98–1.18)	(0.84–1.16)
Concentration of elderly people	0.95 (0.85–1.06)		0.99 (0.89–1.10)	0.86 (0.71–1.01)	0.97 (0.89–1.09)		1.01 (0.92–1.10)	0.97 (0.83–1.10)
Neighborhood social disorganization	1.03 (0.93–1.15)		1.01 (0.91–1.12)	1.14 (0.99–1.28)	1.10* (1.00–1.20)		1.08 (0.98–1.17)	1.18** (1.05–1.31)
Individual SES		1.86**	1.81**	1.87**		1.39**	1.36**	1.38**
		(1.67–2.06)	(1.61–2.00)	(1.67–2.06)		(1.22–1.57)	(1.18–1.54)	(1.20–1.56)
Suburban/urban		1.05				0.82		
		(0.82–1.28)				(0.61–1.03)		
Rural/urban		0.76				0.79		
		(0.45–1.08)				(0.53–1.06)		
Age 40–59/age 20–39		0.77**	0.74**	0.74**		0.76**	0.73**	0.73**
		(0.58–0.96)	(0.55–0.93)	(0.55–0.93)		(0.57–0.95)	(0.55–0.91)	(0.55–0.82)
Age 60+/age 20–39		0.24**	0.23**	0.24**		0.29**	0.28**	0.28**
		(-0.17–0.65)	(-0.17–1.28)	(-0.17–0.65)		(0.04–0.54)	(0.03–0.53)	(0.04–0.53)
Taiwanese		0.63**	0.64**	0.65**		0.71**	0.73*	0.74*
		(0.40–0.86)	(0.41–0.87)	(0.42–0.89)		(0.48–0.94)	(0.50–0.97)	(0.50–0.97)
Married		0.85				0.92		
		(0.66–1.03)				(0.74–1.11)		
Year 1990/year 2000		1.52**	1.51**	1.54**		1.69**	1.68**	1.68**
		(1.27–1.78)	(1.25–1.77)	(1.29–1.80)		(1.47–1.92)	(1.45–1.90)	(1.46–1.91)
Year 1995/year 2000		1.18	1.18	1.21		1.41**	1.41**	1.43**
		(0.91–1.46)	(0.91–1.46)	(0.93–1.48)		(1.19–1.63)	(1.18–1.63)	(1.20–1.65)
Neighborhood education × individual SES				1.17 (0.97–1.37)				1.11 (0.92–1.30)
Concentration of elderly people × individual SES				1.27** (1.09–1.45)				1.07 (0.91–1.24)
Neighborhood social disorganization × individual SES				0.81* (0.63–0.99)				0.83* (0.66–1.00)

*P < 0.05 **P < 0.01 ^a^95% confidence interval

For male drinking, Model 1 shows that men in higher educated and higher socially disorganized neighborhoods were more likely to drink (OR = 1.15; OR = 1.10). Men characterized as higher SES, younger, non-Taiwanese, and interviewed in 1990 and 1995 were more likely to drink than their counterparts. The estimate of neighborhood social disorganization was still statistically significant after including both control variables and interactions of neighborhood education and individual SES (Model 4, OR = 1.18); however, the estimate of neighborhood education was reduced to insignificance in Model 4. A significant interaction was found between neighborhood social disorganization and individual SES (OR = 0.83).

**Figure 1:**
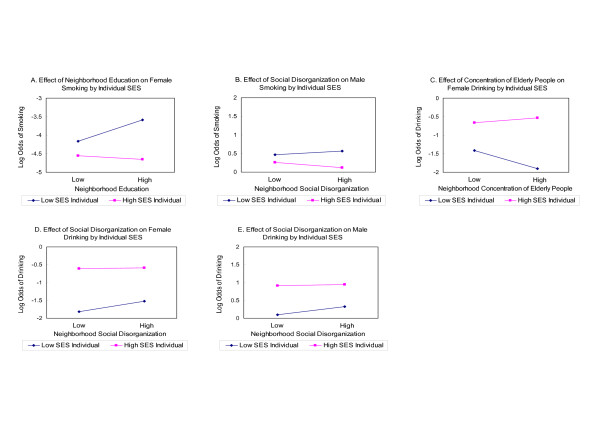
Interactions between neighborhood characteristics and individual SES on smoking and drinking.

Figure 1 presents the interactive relationships found in Table 3 and Table 4. Figure 1A presents the relationship between neighborhood education and female smoking by individual-level SES using a median split. Although low SES women had a higher probability of smoking than high SES women in both low and high educated neighborhoods, the slopes show that neighborhood education has stronger effects for low SES women than for high SES women. In addition, neighborhood education had a strong positive effect on smoking for low SES women, in contrast to a small negative effect on smoking for high SES women. Figure 1B presents the relationship between neighborhood social disorganization and male smoking by individual-level SES. Neighborhood social disorganization had a positive effect on smoking for low SES men, but had a negative effect on smoking for high SES men. Figure 1C and Figure 1D show that neighborhood concentration of elderly people and social disorganization had stronger effects for low SES women than for high SES women. For low SES women, neighborhood concentration of elderly people and neighborhood social disorganization were negatively and positively associated with drinking, respectively. Figure 1E presents the interaction of neighborhood social disorganization and individual SES on male drinking. The slopes show that neighborhood social disorganization was associated with increased drinking only for low SES men.

## Discussion

Since Taiwan government opened the market of tobacco and alcohol to foreign companies in 1987, the society has experienced rises in alcohol use for both men and women. The prevalent rate of male smoking slightly decreased after 1990; however, the prevalent rate of female smoking is increasing [52]. In the last two decades, rapid socio-economic change, such as massive movement of women into the paid work force, alters the role of women in Taiwan [6]. This may result in an increasing use of cigarettes among women. This trend was also reflected in the marketing strategies used by the tobacco companies, in which images of masculine were used in tobacco promotion targeted for the male market, while liberation, glamour, and elite were used for the female market [53,54]. Under this context, this study intends to understand how neighborhood-level factors contribute to the increases of individual smoking and drinking.

Our findings are partly consistent with prior studies that have assessed the associations between neighborhood-level characteristics and individual smoking. Similar to prior studies, we found that higher neighborhood social disorganization was directly associated with higher probability of smoking for women. As documented in Wilson's book *The Truly Disadvantaged *(1987), lower SES neighborhoods where local basic organizations collapse, conventional norms cannot be maintained, high rates of single-parent families are persistent, and high proportions of extremely poor people are isolated from the job network system may increase the likelihood of criminal behaviors and various types of substance abuse. Our study demonstrates that this is the case in Taiwan. Neighborhoods that featured higher rates of single parent families, unemployment, and divorced and separated individuals may have higher rates of female smoking.

Several cross-level interactions were discovered in multilevel analyses. In general, neighborhood characteristics had stronger effects on low SES individuals than on high SES individuals. Low SES individuals may have been more sensitive to local environments as they may have been less knowledgeable about the harmful effects of substance abuse, may have had fewer resources to stop smoking and drinking, and may have experienced more stressors in their daily lives than high SES individuals. We proposed two possible hypotheses, "relative deprivation" and "double jeopardy theory", to explain the interaction between neighborhood environments and individual SES. Our results indicate that different neighborhood dimensions may lead to different interaction patterns. For example, neighborhood education had a positive effect on smoking for low SES women, in contrast to a negative effect on smoking for high SES women. This result supports the hypothesis of "relative deprivation," suggesting that the less educated and less affluent may experience greater levels of stress and anxiety and sharper competition for scarce institutional resources (i.e., access to health care facilities) when competing with better educated, more affluent neighbors. Smoking may be a coping response to stressful neighborhood environments. On the other hand, neighborhood social disorganization seems to have increased effects on drinking behaviors for low SES individuals, but not for high SES individuals. These interactive effects support the hypothesis of the double jeopardy theory, suggesting that living in neighborhoods with high social disorganization will intensify the effects of individual low SES.

Contrary to the findings of previous studies, we found that living in neighborhoods with a higher percentage of elderly people was associated with lower likelihood of drinking for women. Concentration of elderly people was traditionally regarded as a disadvantaged neighborhood characteristic due to lack of community manpower and institutional resources. Most prior studies that examined the effect of concentration of elderly people focused on the outcomes other than smoking and drinking, such as self-rated health and mental health [55]. We are not aware of any study investigating the effects of neighborhood concentration of elderly people on adult smoking and drinking behaviors.

In addition to the explanations of "double jeopardy" and "relative deprivation" hypotheses, the geographical distribution of neighborhoods may partially explain why low SES people in high educated and high social disorganized neighborhoods were more likely to smoke and drink. A large majority of high educated (70%) and high social disorganized (69%) neighborhoods locate in urban areas. Thus the culture of city life may influence one's opportunity to access cigarettes and alcohol. Social gatherings after work are more common in major cities in Taiwan. People relieve stress and renew personal bonds over a drink or by exchanging cigarettes. The provision of alcohol and cigarettes is a way of common courtesy. Low SES people may be more vulnerable to smoking and drinking culture in urban areas as they have fewer personal resources (i.e., knowledge about harmful effects of smoking) to reject smoking and drinking. Future research needs to clarify this relationship by using a better neighborhood geographical typology.

Our findings should be considered in light of the following limitations. First, we did not have longitudinal neighborhood measurements, which may generate selection bias [56]. The relationship between neighborhood characteristics and smoking or drinking may be due to the non-random selection of individuals into neighborhoods and not because of neighborhood influences. Therefore, these relationships should be interpreted as associations only. Second, we did not measure the length of time that participants had spent in their neighborhoods and the extent of their exposure to the neighborhood environment. We were thus unable to determine whether effects of neighborhood characteristics on smoking and drinking behaviors were due to cumulated effects [56]. Third, we did not measure all social and physical aspects of neighborhoods, such as informal social control, concentration of tobacco and alcohol outlets, and availability of social service agencies [47]. Future research needs to improve neighborhood measurements by assessing multiple aspects of neighborhoods. Fourth, in contrast with most prior studies that measure the outcomes of problem drinking behaviors (i.e., binge drinking or drunk driving), our study identified drinkers if they had ever used alcohol. Because we do not have enough cases of problem drinking, we were unable to assess the neighborhood influences on problem drinking behaviors. Nevertheless, the way that we categorized alcohol use into "no use" and "at least some" can examine how neighborhood characteristics affect one's opportunities of exposure to any alcohol or one's decision to be a non-drinker. Fifth, we calculated the employment rate by the number of employed people divided by the population aged 15 over; however the employment rate should be calculated by the number of employed people divided by the size of labor force, which excludes housewives, students, disabled, and retired persons. Because the 2000 Taiwan census survey did not clarify the reasons why people were unemployed, we were not able to assess the impact of neighborhood employment rate on participants' behaviors. The impact of this inappropriate measurement may be limited, though, because the employment rate only contributes partially to the measurement of neighborhood social disorganization.

## Conclusion

This study demonstrates the importance of examining neighborhood influences on smoking and drinking behaviors in Taiwan. Our findings suggest that neighborhood characteristics may influence individual smoking and drinking directly and interactively with individual SES. Future neighborhood research is needed to identify possible mechanisms by which neighborhoods can influence one's smoking and drinking behaviors in a context of Asian society.

## Competing interests

The author(s) declare that they have no competing interests.

## Authors' contributions

YC has contributed to designating the study, acquiring the data, conducting the statistical analysis, interpreting the empirical analysis and preparing the manuscript. YL and YW have contributed to acquiring the data, conducting the statistical analysis, and interpreting the empirical analysis. HJC has contributed to interpreting the empirical analysis and preparing the manuscript.

## Pre-publication history

The pre-publication history for this paper can be accessed here:


